# IsiZulu-speaking caregivers’ perceptions of child language stimulation

**DOI:** 10.4102/sajcd.v71i1.1028

**Published:** 2024-07-25

**Authors:** Desiree C. John, Catharina J. Uys, Michelle Pascoe

**Affiliations:** 1 Discipline of Speech-Language Pathology, College of Health Sciences, University of KwaZulu-Natal, Durban, South Africa; 2 Department of Occupational Therapy, Faculty of Health Sciences, University of Pretoria, Pretoria, South Africa; 3 Department of Health and Rehabilitation Sciences, Faculty of Health Sciences, University of Cape Town, Cape Town, South Africa

**Keywords:** beliefs, caregiver perceptions, cognitive-linguistic development, culture, early language stimulation, speech-language therapy, South Africa

## Abstract

**Background:**

South African children from low-income households are at higher risk of cognitive-linguistic difficulties because of multiple risk factors. Early language stimulation minimises the effects of risk factors and prepares children for literacy and learning. Understanding caregivers’ perceptions of language stimulation is important because perceptions shape practices, which determine child language outcomes.

**Objectives:**

This study explored the existing perceptions of language stimulation among 15 isiZulu-speaking caregivers from KwaDabeka township.

**Method:**

A qualitative, descriptive research design was adopted and the caregivers were interviewed using a semi-structured interview schedule. The NVivo software programme supported the inductive, thematic analysis of the data.

**Results:**

Although the caregivers held positive perceptions of language stimulation, their perceptions of benefit focussed on meeting children’s basic physiological needs with less reference to the long-term benefits for literacy, employment and social integration. Providing language models, watching television, singing songs, and reading books were perceived to be examples of language-stimulating activities and techniques. The caregivers expressed a need to improve their knowledge of language stimulation and queried how they could be supported to achieve this outcome.

**Conclusion:**

This group of caregivers needed support to increase their awareness of the long-term benefits of language stimulation and their knowledge and use of evidence-based activities, stimuli and facilitation techniques.

**Contribution:**

Caregivers’ existing perceptions could serve as a barrier to the effective language stimulation of children from low-income households in South Africa.

## Introduction

South African children from low-income households are at higher risk of cognitive-linguistic difficulties because of multiple risk factors (Hall et al., 2018). The primary factors that have threatened the development of such children include undernutrition, family stress, diseases, violence, trauma and substance abuse (Chakona, 2020). Early language difficulties have pervasive and long-term effects on children’s development and well-being (Spaull & Hoadley, 2018). Some studies, for example, showed how spoken language proficiency predicted reading development (Hulme & Snowling, 2016; Russell et al., 2018). Other studies demonstrated the relationship between language development, psychosocial functioning and mental health (Saugestad et al., 2022). The relationship between language abilities and academic performance has also been well-documented (Aguilar-Mediavilla et al., 2019; Kaiser et al., 2022). Hence, children who are at higher risk of cognitive-linguistic difficulties may need support to develop their spoken language proficiency before school entry.

All children, irrespective of their ethnic identity, need to be nurtured to reach their full linguistic potential (Mesman et al., 2012). Caregiver-child communicative interactions therefore serve as the critical context for early language learning (Heidlage et al., 2020). Even at a preverbal level, caregiver input contributed to language development by translating the child’s gestures into language (Romano & Windsor, 2020). Another study demonstrated how caregiver input mediated the effect of socioeconomic status on child language development (Biel et al., 2020). Caceres et al. (2016) believed that supporting caregivers is one of three principles that could strengthen the existing policies on child development. The other two principles were supporting child development from birth and making resources available to the most vulnerable. It is concluded that enabling caregivers to stimulate their children is one of the keys to promoting early language development in South Africa.

Exploring caregivers’ perceptions of language stimulation is significant because they determine language stimulation practices and child language outcomes. Liew et al. (2018) showed how childrearing perceptions were precursors to parenting practices and children’s psychosocial and academic outcomes. Another study reported on parents’ perceptions of self-efficacy and the influence it had on their practices (Glatz & Trifan, 2019). Habibi et al. (2017) demonstrated how parents with accurate perceptions selected stimuli appropriately and stimulated their children more frequently. Zellman et al. (2014) reported that parents with positive attitudes and higher levels of parenting efficacy had children who were better readers at ages 6 and 7 years. Finally, another study showed how parents with more accurate perceptions of their children’s development had children who behaved better in the latter years (Chung et al., 2019). These findings suggest that the relationship between caregivers’ perceptions, their practices and children’s language outcomes cannot be underestimated.

Age, ethnicity, education and socioeconomic status are some of the variables, which influence a caregiver’s perceptions of language stimulation. Culture, for example, conditioned perceptions and perceptions shaped behaviour (Kastanakis & Voyer, 2014). Caregivers with a higher socioeconomic status had more accurate perceptions of their children’s development (Chung et al., 2019). Parents’ perceptions mediated the relationship between maternal education and children’s language and literacy outcomes (Rowe et al., 2016). Moreover, education influenced parent’s perceptions when ethnicity was controlled and ethnicity influenced parent’s perceptions when education was controlled (Rowe et al., 2016). Caregiver mental health influenced caregivers’ perceptions of infant development in a South African study (Bain & Richards, 2016). These findings confirm that multiple variables could shape a caregiver’s perceptions of language stimulation.

### Caregivers’ perceptions of language stimulation

Caregivers had distinct perceptions about the different domains of child development as they knew more about motor and socio-emotional development and less about cognitive-linguistic development. This finding was observed in caregivers from South African families, Asian families and even some families from the United States of America (US) (Campbell et al., 2019; Habibi et al., 2017; Van Belkum & Meintjes, 2013). While culture was cited as one of the reasons for this, motor development was perceived to be more overt and less abstract than speech and language development (Van Belkum & Meintjes, 2013). Chung et al. (2019) warned that a lack of knowledge or misperceptions could have long-term negative consequences for child development. Thus, caregivers’ perceptions of the different domains of child development could result in some domains of development receiving more attention than others.

Caregivers’ views varied with regard to the benefits of early language stimulation. A study by Williams et al. (2014) revealed that caregivers understood the relationship between early language stimulation and later academic development. Another study revealed that parents did not believe that the early years were significant as children did not remember what happened to them when they were young (Zellman et al., 2014). A cohort of South African caregivers perceived that early stimulation was important but identified early to be between 3 and 6 years of age or when children were verbal (Richter et al., 2019). A caregiver’s perceptions of benefit are significant because they determine if caregivers will stimulate their children, when they will commence language stimulation and if they will persist in the face of no immediate or obvious change in language development.

Caregivers’ perceptions also varied with respect to the role that they played during language stimulation. Some caregivers believed that development was malleable and parents played an instrumental role in their children’s language development (Chung et al., 2019; Williams et al., 2014). The caregivers from another study reported that speaking was inherited so all late talkers will eventually begin to speak on their own (Habibi et al., 2017). A study conducted by Vahdat et al. (2016) revealed that 14% of caregivers corrected their children’s language errors. While acknowledging the instrumental role that they played during stimulation, South African caregivers believed that it was a shared responsibility (Richter et al., 2019). Sharing the childrearing responsibility is typical of interdependent or collectivist cultures (Lynch & Hanson, 2011).

Caregivers had different views on effective language learning activities. The caregivers from one study believed that reading (63%), talking (55%) and singing (50%) promoted language development (Williams et al., 2014). A healthy diet scored the highest percentage for influencing cognitive-linguistic development over other activities such as reading, talking and playing (Zellman et al., 2014). Wadende et al. (2016) stated that household chores, storytelling and singing were indigenous activities, which rural Kenyan children found intrinsically motivating. A South African study revealed that young children were stimulated during household chores, daily routines, play and singing (Earl, 2011). Hence, effective activities might be those that are perceived to be intrinsically motivating for the caregiver and the child.

Caregivers have distinct preferences for certain types of language learning materials. A group of caregivers from four different nations preferred traditional toys (blocks and animals) over digital toys and games (Isikoglu Erdogan et al., 2019). Similarly, caregivers from Hong Kong chose print books instead of e-books for their children (Sung & Chiu, 2022). Other caregivers, however, preferred to expose children to digital learning materials from a young age to prepare them for later use (Radesky et al., 2016). While caregivers have specific preferences, extrinsic factors such as child safety, physical space and affordability determined children’s exposure to language learning materials (Balton et al., 2019). Moreover, intrinsic factors such as caregiver reading difficulties or discomfort with reading influenced children’s exposure to print media (Justice et al., 2015). Thus, children’s exposure to language learning materials is influenced by many factors.

Modelling language emerged as the common language facilitation strategy across studies. One study revealed that Australian mothers preferred indirect language facilitation strategies (modelling language) instead of direct facilitation strategies (asking questions) (Williams et al., 2014). Two South African studies also reported caregivers’ preferences for modelling language to support the development of young children (Earl, 2011; Pascoe et al., 2016). Moreover, other techniques, such as imitation and expansion of children’s utterances scaffolded the language development of older children. Nevertheless, Duranti et al. (2012), reported that there were few opportunities for modelling, imitation and expansion in non-dyadic patterns of communicative interaction. Although modelling language emerged as the dominant facilitation technique, the way the communicative interaction is structured may influence the choice of facilitation techniques.

Finally, caregivers’ perceptions of their support needs have also been documented in the literature. Studies have indicated that caregivers want to know more about cognitive-linguistic development (Van Belkum & Meintjes, 2013; Williams et al., 2014). Caregivers from under-resourced contexts preferred to be supported by healthcare professionals (Jang & Beck, 2018). The support should be provided in the second 6 months of the child’s life, as families need time to adjust to the new addition (Arora et al., 2018). Supporting groups of caregivers was preferred over individual support and observation and demonstration were the chosen learning methods (Gboku et al., 2007). Parent manuals, handouts and videos were the selected learning materials (Heidlage et al., 2020; Rajesh & Venkatesh, 2019).

### The rationale for the study

South African children from low-income households are at higher risk of cognitive-linguistic difficulties because of multiple risk factors. Early language stimulation mitigates the effects of risk factors and prepares children for literacy and learning. Understanding caregivers’ perceptions of language stimulation is important because perceptions influence practices, which determine child language outcomes.

## Research methods and design

### Aim

This study explored a group of isiZulu-speaking caregivers’ perceptions of stimulating child language development in KwaDabeka township in the province of KwaZulu-Natal.

### Research design

A qualitative, descriptive research design was adopted as it aimed to explore participants’ perspectives on stimulating child language development (Doyle et al., 2020). While exploring participants’ perspectives of phenomena is also true of other qualitative designs, descriptive designs are differentiated by the fact that they stay close to the data. Staying close to the data means that they do not seek to provide rich descriptions as in ethnographic research, deep interpretations as in phenomenological research or theoretical explanations as in grounded theory research (Doyle et al., 2020). The theoretical and philosophical underpinnings of a descriptive design are, however, similar to other types of qualitative research as it draws from constructionism, critical theory and pragmatism. This design was also selected because of its suitability for addressing issues of clinical significance in healthcare practice settings (Pelentsov et al., 2016).

### Study context

KwaDabeka is a peri-urban township that is situated 22 kilometres west of Durban. Most of the residents are African (99.6%) whose home language is isiZulu (88%) (Statistics South Africa, 2011). The community accesses healthcare from a primary healthcare clinic and outreach programmes. Registered creches and preschools support the development of some of the young children in the community. Some of the challenges faced by peri-urban townships are higher unemployment levels and lower education and income levels (Mahajan, 2014). Female-headed households (43.2%) are most vulnerable, as many young mothers do not return to school after they have given birth; resulting in many families relying on social grants to survive. Moreover, only 6% of the people living in townships had tertiary education or vocational training (Mahajan, 2014). The literacy rates of learners who resided in townships were lower than the rates in urban and suburban areas in South Africa (Howie et al., 2017). Access to healthcare is influenced by traditional beliefs even though the burden of disease is higher among poor people.

### Participants

The participants were recruited from the immunisation clinic at the community health centre in the township. The researcher and a qualified interpreter explained the nature and purpose of the research and extended the invitation to the caregivers to participate in the research. Interested caregivers were given an appointment to meet the research team at a non-governmental organisation (NGO) within the community. Homogenous, purposive sampling was used to select 15 caregiver-child dyads. The caregivers were included in the study if their home language was isiZulu, if they had a minimum education level of Grade Eight, and if they were the primary caregivers of the children. The children were excluded if they were identified as having a severe developmental disability. This was determined by administering a screening tool, which included two components: a parent report on child development and a review of the immunisation records for risk status (Ertem et al., 2008; The South African National Department of Health, 2020).

The parent report on child development was based on the Guide for Monitoring Child Development (Ertem et al., 2008). This screening tool has been used to detect children (0–2 years) with developmental disabilities in under-resourced contexts. It contains items that are not culture-specific and cover the following developmental domains: receptive and expressive language, gross and fine motor development, socio-emotional development and play. Cognition is evaluated through the domains of play and language. Sensitivity and specificity measures suggested that the tool is appropriate for detecting developmental disabilities in young children from South Africa (Ertem et al., 2019). Furthermore, the child’s Road to Health Book was reviewed for Appearance, Pulse, Grimace, Activity and Respiration (APGAR) scores, birth weight and record of pregnancy and birth complications (The South African National Department of Health, 2020, p. 38). The researcher and the research assistant, who was a bilingual speech-language therapist, were responsible for screening the children.

The chronological age of the caregivers and the children ranged from 23 to 38 years and 9 to 22 months, respectively. All the caregivers were female and their educational levels ranged from Grade 10 to 12. Thirteen caregivers were registered for the MomConnect Programme, which is an initiative of the South African National Department of Health (Trafford et al., 2020). This programme, which relies on cell phone-based technology, sends health promotion messages via SMS or WhatsApp to strengthen maternal and infant health and improve survival rates. As 63% of South African mothers are registered for this programme, the caregivers in this study represented the majority of mothers who accessed public health facilities in South Africa (Trafford et al., 2020). All the caregivers were the biological parents of their children.

### Data collection

The data were collected by conducting semi-structured in-person and telephonic interviews. In-person interviews were conducted outside of the global pandemic while telephonic interviews were conducted during the global pandemic because of reduced access to the community and the restrictions placed by the research ethics committee. The same interview schedule was used for both interviews and the two types of transcripts were evaluated for differences in depth and scope of data. The interview schedule was developed according to the five steps outlined by Waltz et al. (2016), which included a review of the literature, formulation and sequencing of the questions and drafting and piloting of the schedule. A content expert (a bilingual speech-language therapist) viewed the draft schedule and provided written feedback. Thereafter, the draft schedule was piloted on five isiZulu-speaking caregivers who met the key criteria for inclusion to the study. The researcher and a qualified interpreter were responsible for the piloting of the interview schedule.

The draft schedule was edited to add, remove or change questions and the pilot transcripts were reviewed for depth and scope of information. The final interview schedule contained the instructions for the interviewer, an opening statement for the participant, the interview prompts and a closing statement for the participant. The interviews were conducted by the researcher and another bilingual speech-language therapist. The researcher, who is an academic employed at a university, trained the assistant to conduct the interviews. The training consisted of a demonstration by the researcher, practice and feedback. A Sony ICD-PX312 voice recorder was used to record the in-person interviews while the Call Recorder-Cube ACR recording application was used to record the telephonic interviews. The in-person interviews were conducted in a side hall, which was separate from the rest of the NGO. The side hall provided a safe, quiet and private space for the caregivers. Table 1 contains the English interview prompts.

**TABLE 1:** The interview prompts: Caregiver perceptions of stimulating child language development.

**Purpose** To explore the caregivers’ perceptions of stimulating child language development.
**Opening prompt** What would you like your child to learn as he or she grows?
**Key prompt 1** Is learning to talk an important milestone for your child?
**Associated prompts 1** Why is learning to talk important? How did your child learn to talk? Did you help your child to learn how to speak? At what age did you start helping him or her? What advice would you give to other mothers who want to help their children to talk?
**Key prompt 2** Do you need to learn more about your child and talking?
**Associated prompts 2** What would you like to learn? How would you like to learn this? When and where would you like to learn? Who should teach you? What would make it difficult for you to learn? What words or sentences would you like your child to learn?
**Closing prompt** Is there anything that you would like to add on the topic of children and talking?

### Data analysis

The voice recordings were transcribed onto a template with the following column headings: person and utterance. The transcripts were translated into English by the bilingual speech-language therapist and were uploaded to the NVivo 12 Pro software programme for analysis (https://www.qsrinternational.com). The researcher conducted an inductive thematic analysis, which developed theory from the data through the process of inductive reasoning. This contrasts with deductive thematic analysis that uses theory to inform the data analysis (Braun & Clarke, 2006). Two coding lenses were applied during the analysis. Firstly, value coding converted the raw data into codes. Value coding was appropriate for this study as caregiver perceptions may be influenced by cultural beliefs and values (Kastanakis & Voyer, 2014; Saldana, 2016). Then, pattern coding converted the codes into categories and themes. Pattern coding is the synthesis of a large number of codes into a significant whole (Saldana, 2016).

### Ethical considerations

Ethical recertification was obtained from the University of KwaZulu-Natal Biomedical Research Ethics Committee (BFC 568/17) on the 17 October 2023. Informed consent was obtained by orientating the caregiver to the research verbally and then giving the caregiver the written consent form to discuss with other family members before signing it. The research assistants signed declarations of confidentiality and codes were applied to conceal participants’ identities. The research team acknowledged the influence of power and authority when engaging with the participants. Effective communication (written scripts and visual schedules) and increased caregiver decision-making were two strategies that were applied to minimise the differences. Voluntary participation was highlighted and the participants were orientated as to how the data will be stored and used. Participants were treated with justice and equity.

### Data quality

The following steps were taken to enhance the trustworthiness of the data. The research assistants were bilingual speech-language therapists who understood the culture and the language of the participants. Training was provided to achieve data collection and analysis outcomes. Weekly meetings with the research assistant provided opportunities for reflection on the research process and findings. Written scripts of each step in the data collection process regulated the data collection procedures across participants. Visual schedules supplemented oral explanations to reduce linguistic barriers during the in-person interviews (http://goboardmaker.com).

## Results and discussion

Three main themes emerged after the thematic analysis of the data. These themes captured a plausible explanation of the caregivers’ perceptions of stimulating child language development. Tables and participant text are included to validate the findings. The text is preceded by the participant code, for example, CG14 represents caregiver 14.

### Theme 1: Positive perceptions of language stimulation

Most of the caregivers held positive perceptions of language stimulation, which was therefore perceived to be beneficial. While the caregivers cited many benefits, meeting the children’s basic physiological needs (safety, hunger, thirst) was the immediate priority. Although most of the caregivers identified social integration as one of the primary parenting goals, there were few references to communication for social integration. Moreover, few caregivers referred to the relationship between language stimulation, literacy, learning and employment. None of the caregivers referred to the important role that the first language plays in the acquisition of a second language. Table 2 illustrates the number of caregivers and reference frequency for some of the codes for theme one.

**TABLE 2:** Sample of codes for theme one.

Codes	NoC	FoR	Participant number	Participant response	English translation
**Category: Benefits of language stimulation**					
Communication for meeting basic physiological needs	12	20	CG8	*‘… ukuze ezongitshela uma ngabe egula.’*	‘… so that she will tell me when she is sick.’
Communication for social integration	2	2	CG12	*‘…ukuze ukwazi ukuxhumana nabanye abantu.’*	‘… so that he can communicate with other people.’
Communication for education	1	1	CG2	*‘… kuzobasiza ukuthi bakwazi ukufunda esikoleni.’*	‘… at school it will help them to be able to learn.’
Communication for employment	1	1	CG5	*‘Bazokwazi ukuthi baphendule yonke into abayibuzwayo.’ (kwinhlolokhono)*	‘They can be able to answer everything that they are asked.’ (in an interview)

Note: NoC refers to the total number of caregivers who produced text that met the NVivo software code’s definition or description. FoR refers to the total number of references per NVivo code. A caregiver could, for example, refer to a code more than once.

Abbreviations: FoR, frequency of reference; NoC, number of caregivers.

The children in this study were young and this could explain why the caregivers’ perceptions of benefit were focussed on meeting children’s basic physiological needs. However, if a child’s language skills at 2 years predicted language development at 4 and 5 years of age (Gardner-Neblett & Iruka, 2015), caregivers from under-resourced contexts need to understand the long-term benefits of language stimulation from early on. Awareness of the long-term benefits could encourage caregivers to stimulate their children from an early age and be persistent and consistent in their efforts, as the effects of language stimulation may not be immediate or obvious. The findings of this article were similar to the findings of the Zellman et al.’s (2014) study as both groups of caregivers did not refer to the relationship between early language development and later learning. This was in contrast to the findings of the Williams et al. (2014) study, where the caregivers held clear perceptions of this relationship.

### Theme 2: Perceptions of effective practices

Most of the caregivers indicated that they should be the primary facilitators of the children’s language. Other family members and early childhood education workers played a secondary role. Some of the caregivers felt that language stimulation should commence before the child turned 1 year old and before the onset of the first words. Modelling language was the dominant facilitation technique while watching television, singing songs, and reading books were cited to be effective activities. Although reading books was perceived to be effective, children were exposed to magazines and newspapers, which suggested that there was a need for more appropriate reading materials in the home. Television was the main type of electronic media that was used in the home. Table 3 illustrates the number of caregivers and reference frequency for some of the codes for theme two.

**TABLE 3:** Sample of codes for theme two.

Codes	NoC	FoR	Participant number	Participant response	English translation
**Category: Language facilitators**					
Primary caregiver as a language facilitator	12	34	CG14	*‘Kuwumsebenzi kamama.’*	‘It’s the mother’s responsibility.’
Other family members as language facilitators	5	6	CG11	*‘Ufunde ngokuthi alalele ugogo wakhe.’*	‘He learnt by listening to his granny.’
ECE worker as a language facilitator	3	5	CG3	*‘… ngakho ke, lokho kungasho ukuthi baqala ukufunda enkulisa.’*	‘… so, that would mean that they are catching on from preschool.’
**Category: Language facilitation techniques**					
Model language	7	7	CG11	*‘Ukukhomba izinto bese eyasho ukuthi ziyini.’*	‘To point at things and say what they are.’
Correct errors	3	3	CG15	*‘Ngiyamlungisa.’*	‘I correct her.’
Give instructions	1	1	CG9	*‘Amucele ukuthi ayohaga ugogo.’*	‘Ask him to go hug granny.’
**Category: Language stimulation activities**					
Watch television	7	13	CG1	*‘… ngokubuka i-TV … ngokulalela i-radio (umsakazo).’*	‘… by watching TV … by listening to the radio.’
Sing songs	7	11	CG14	*‘Culela ingane.’*	‘Sing to the child.’
Reading	4	8	CG1	*‘Bayakwazi nokubona izithombe.’*	‘They can also see the pictures.’
Caregiving routines	2	4	CG9	*‘Ngikhuluma naye ngesikhathi ngimugeza … nalapho ngimufunza.’*	‘I speak to him during bathing … when I am feeding him.’

Note: NoC refers to the total number of caregivers who produced text that met the NVivo software code’s definition or description. FoR refers to the total number of references per NVivo code. A caregiver could, for example, refer to a code more than once.

Abbreviations: ECE, early childhood education worker; FoR, frequency of reference; NoC, number of caregivers.

Although viewing television programmes emerged as one of the effective practices, the literature is inconclusive regarding the benefits of using electronic media for the language learning of young children (Medawar et al., 2023). Leung et al. (2020), for example, reported that young children are more likely to learn new words from contact with other people rather than watching television, as the passive viewing of television has a low impact on language development. Although the literature classifies shared book reading as an effective evidence-based practice (Van Kleeck, 2006), the caregivers in this study needed more appropriate reading materials to facilitate this activity successfully. Few reading materials were also cited as a barrier in other South African studies (Coetzee et al., 2023; Tayob & Moonsamy, 2018). The findings suggested that this group of caregivers needed support to identify appropriate activities and to access appropriate materials for language stimulation.

Modelling language emerged as the primary facilitation technique in this study. This was consistent with the findings from two other South African studies, which cited modelling language as the key strategy (Earl, 2011; Pascoe et al., 2016). The literature, however, highlights other techniques that are also effective for the language stimulation of young children (Newman et al., 2016). A scoping review of 21 studies revealed that imitation and expansion of children’s utterances were effective strategies (Akamoglu & Meadan, 2018). Imitation is where the caregiver copies the child’s previous utterance without adding new information and this contrasts with expansion, which is the addition of new words or vocalisations (Roberts et al., 2014). Hence, expanding the range of facilitation techniques could support this group of children’s language development better.

### Theme 3: Perceptions of support needs

The caregivers expressed a need to improve their knowledge of language stimulation and shared how they could be supported to achieve this outcome. They wanted to be trained by healthcare professionals or people who were knowledgeable about children and talking. The caregivers preferred a combination of contact learning in a group and individual, self-directed learning in the home. Videos were the preferred learning resources. Written materials such as books and pamphlets were the second option. A positive attitude towards learning about language stimulation was the primary facilitator. Insufficient time to learn was identified as the primary barrier to learning as some of the caregivers were employed outside of the home, were single mothers or were students. Table 4 illustrates the number of caregivers and reference frequency for some of the codes for theme three.

**TABLE 4:** Sample of codes for theme three.

Codes	NoC	FoR	Participant number	Participant response	English translation
**Category: Caregiver learning needs**					
How to stimulate language	7	7	CG14	*‘Ngakho ke, ngifuna ukwazi ukuthi ngithini kanye nokuthi nini.’*	‘So, I want to know what to say and when.’
Bilingual development	6	7	CG2	*‘Ukufunda izilimi ezimbili.’*	‘Learning two languages.’
**Category: Caregiver learning materials**					
Videos	7	7	CG14	*‘Ngingathanda amavidiyo.’*	‘I would like videos.’
Written material	6	10	CG7	*‘Izincwadi nezinto ezinjalo.’*	‘Books and things like that.’
**Category: Facilitators of caregiver learning**					
Positive attitude	10	11	CG7	*‘Udinga ukwazi.’*	‘You need to know.’
Time	7	8	CG5	*‘Ngithole isikhathi ngoba uya enkulisa.’*	‘I got time because she goes to preschool.’
**Category: Barriers to caregiver learning**					
Time	9	11	CG9	*‘Isikhathi singaba inkinga kimi’*	‘Time would be a problem for me.’
Transport costs	1	1	CG8	*‘… nasendaweni engingakwazi khona ukukhokhela izimoto zokuhamba.’*	‘… and at a place where I can afford transport.’

Note: NoC refers to the total number of caregivers who produced text that met the NVivo software code’s definition or description. FoR refers to the total number of references per NVivo code. A caregiver could, for example, refer to a code more than once.

Abbreviations: FoR, frequency of reference; NoC, number of caregivers.

The caregivers preferred to be trained by a healthcare professional, which is consistent with previous research conducted by Jang and Baek (2018). A positive attitude towards learning about language stimulation was consistent with the findings from the study conducted by Van Belkum and Meintjes (2013), where 93% of the caregivers were willing to implement a stimulation programme in the home. Insufficient time was also identified as the primary barrier in other studies; especially if the caregiver was employed outside of the home (Rostad et al., 2018). Learning in groups and with videos is consistent with the principles of African pedagogy (Serpell & Adamson-Holley, 2016). As speech and language are abstract concepts, it is recommended that speech-language therapists support caregivers to identify their needs with regard to stimulating children’s language development.

Some of the limitations of the study methodology are discussed next. Although it was unavoidable, some of the interviews were conducted in person while other interviews were conducted telephonically and this could have affected the quality of the data. Although several steps were taken to protect the integrity of the data (training research assistants, using written scripts and visual schedules), meaning could have been altered or lost during the cross-cultural and cross-linguistic research process. The differences in the social status between the research team and the participants affected the participants’ responses during data collection.

## Conclusion

The current research explored a group of isiZulu-speaking caregivers’ perceptions of stimulating child language development. The rationale for exploring the caregivers’ perceptions was based on the intricate relationship between caregivers’ perceptions, language stimulation practices and child language outcomes. The findings from this study suggested that this group of caregivers needed support to meet the language stimulation needs of their children. Three specific areas were highlighted. Firstly, the caregivers’ awareness of the long-term benefits of language stimulation could be increased as this could motivate caregivers to stimulate their children’s language development from an early age. Secondly, the caregivers could be supported to identify evidence-based activities and materials for language stimulation. Thirdly, the caregivers could be supported to expand the range of facilitation techniques used during language stimulation. Most of the findings of this study were consistent with previous research on caregivers’ perceptions of language stimulation.

It is concluded that caregivers’ perceptions could serve as a barrier to the effective language stimulation of children from low-income households. Socially valid preventative interventions, which address caregivers’ perceptions and practices, could support caregivers to meet the language stimulation needs of their children. Such interventions could minimise the effects of secondary complications and prepare children for literacy and learning. Speech-language therapists, therefore, play a critical role in the development, implementation and evaluation of socially valid preventative interventions, which constitutes one of the major implications for future research.
